# Extracellular fluid volume: A suitable indexation variable to assess impact of bariatric surgery on glomerular filtration rate in patients with chronic kidney disease

**DOI:** 10.1371/journal.pone.0256234

**Published:** 2021-08-16

**Authors:** Caroline Grangeon-Chapon, Audrey Laurain, Vincent L. M. Esnault, Coralie Cruzel, Antonio Iannelli, Guillaume A. Favre

**Affiliations:** 1 Department of Nuclear Medicine, University Hospital of Nice, Archet 1 Hospital, Nice, France; 2 Department of Pharmacy, University Hospital, Nice, France; 3 Department of Nephrology, University Hospital, Pasteur 1 Hospital, Nice, France; 4 Centre National de la Recherche Scientifique, UMR 7073, Laboratory of Physiology and Molecular Medicine (LP2M), Nice, France; 5 University of Côte d’Azur, Nice, France; 6 Department of Clinical Research and Innovation, University Hospital, Cimiez Hospital, Nice, France; 7 Digestive Surgery and Liver Transplantation Unit, University Hospital of Nice, Archet 2 Hospital, Nice, France; 8 Inserm, U1065, Team 8 “Hepatic complications of obesity and alcohol”, Nice, France; University of Colorado Denver School of Medicine, UNITED STATES

## Abstract

**Background:**

Bariatric surgery (BS) might be a nephroprotective treatment in obese patients with chronic kidney disease (CKD), and the non-linear relation between body surface area (BSA) and extracellular fluid volume (ECFV) in obese people raises the question of the most relevant way to scale glomerular filtration rate (GFR) for assessing renal function changes after BS.

**Methods:**

We screened 1774 BS candidates and analysed 10 consecutive participants with CKD stage 3. True GFR (mGFR), measured by the renal clearance of ^51^Cr-ethylenediaminetetraacetic acid (EDTA), was scaled either to BSA (mGFR_BSA_) or to ECFV measured by ^51^Cr-EDTA distribution volume (mGFR_ECFV_) before and one year after BS.

**Results:**

The 10 candidates for BS had a mean body mass index of 43.3 ± 3.6 kg/m^2^ and a mean GFR of 48 ± 8 mL/min/1.73 m^2^. Six participants had a sleeve gastrectomy and four had a Roux-en-Y gastric bypass. One year after BS, ECFV decreased (23.2 ± 6.2 to 17.9 ± 4.3 L, p = 0.001), absolute mGFR was not significantly modified (74 ± 23 versus 68 ±19 mL/min), mGFR_BSA_ did not change significantly (53 ± 18 versus 56 ± 17 mL/min/1.73 m^2^) whereas mGFR_ECFV_ significantly increased (42 ± 13 versus 50 ± 14 mL/min/12.9 L, p = 0.037). The relation between mGFR_ECFV_ and mGFR_BSA_ was different from the identity line before (p = 0.014) but not after BS (p = 0.09).

**Conclusion:**

There is a difference between mGFR_BSA_ and mGFR_ECFV_ following BS and the latter might better reflect the adequacy between renal function and corpulence.

## Introduction

In the United States, 7.4 million people with chronic kidney disease (CKD) are deemed to be severely or morbidly obese [1], and about 13,000 of them were waitlisted for kidney transplantation between 1995 and 2006 [2]. Bariatric surgery (BS) is the most effective strategy for a sustained long-term weight loss in morbidly obese individuals. BS might contribute to nephroprotection because it reduces glomerular hyperfiltration, which is an independent factor of renal function loss [3], improves the adipocytokines profile [4] and induces the remission of hypertension and type 2 diabetes (T2D) [5]. A randomized control trial is ongoing to analyse the trajectory of the renal function after BS in obese patients with CKD stage 3 or 4 (BOKID, ID-RCB 2014-A011884-43).

Estimation of glomerular filtration rate (eGFR) is biased in obese patients undergoing BS because creatinine and cystatin C productions decrease following lean and fat mass losses [6]. Therefore GFR changes before and after BS can only be assessed by the measurement of true GFR (mGFR) using the clearance of an exogenous filtration tracer. So far, only one study analysed mGFR changes after BS using the renal clearance of ^125^I-iothalamate in a cohort of 13 individuals with CKD stage 3 or 4. Absolute mGFR (mL/min) remained unchanged, suggesting the presence of a poor functional renal reserve [7]; mGFR scaled to body surface area (mGFR_BSA_, mL/min/1.73 m^2^) increased following weight loss [8]. However, the indexation of GFR by BSA may be irrelevant here because the relation between BSA and the extracellular fluid volume (ECFV) is not linear in obese individuals [9–11]. Several authors have already shown that indexing for 1.73 m^2^ body surface area would be a mistake for assessing GFR after BS [12,13]. The purpose of the present study is to compare mGFR_ECFV_ and mGFR_BSA_ before and one year after surgery. This study was powered to show a difference between mGFR_ECFV_ before and one year after BS in obese individuals with CKD stage 3.

## Materials and methods

Candidates to BS were recruited in our tertiary referral bariatric center. All patients meeting the criteria for BS [14] were invited to take part in this study only if their estimated GFR based on serum creatinine using the MDRD formula (Modification of Diet in Renal Diseases) was less than 60 mL/min/1,73m^2^ twice at three months interval.

The following data were recorded before and one year after BS: age, sex, height, obesity-related comorbidities (T2D, hypertension, sleep apnea syndrome, disabling osteoarticular disease, non-alcoholic fatty liver disease), weight, waist circumference, body mass index (BMI), presence or absence of physical signs of edema, number of anti-hypertensive drugs, urinary albumin over creatinine ratio (UACR), daily urinary creatinine output, plasma leptin level, measured GFR and measured ECV. Weight loss was reported as percent of excess BMI loss and calculated as follows [15]:

%EBMIL=[(BMIbeforesurgery−finalBMI)/(BMIbeforesurgery−25)]×100

Lean and fat mass were estimated before and one-year after BS by the 24h urinary creatinine output and by the plasma level of leptin respectively.

GFR was measured by the renal clearance of ^51^Cr-ethylenediaminetetraacetic acid (EDTA) and ECV by the distribution volume of ^51^Cr-EDTA with successive plasmatic and urinary samples collection every 15 minutes. Participants received a bolus injection of 22 kBq/kg (0.6 μCi/kg) then a constant infusion hourly rate of 7 kBq/mL.min^-1^.1.73m^-2^ of eGFR (0.19 μCi/mL.min^-1^.1.73^−2^). The first urinary collection was obtained at 1h15 after bolus injection. The mGFR was determined by calculating the glomerular clearance (Cl) from plasma concentrations (P) and renal excretions per unit of time (U x V) during infusion at a constant plasma level of the tracer (Cl = U x V/P). The result was given as mean value of several consecutive clearances. Because correct urine collection is a key for the procedure, seven collection periods (instead of 5 usually) were performed for the calculation of the mean clearance. For each collection period, ECV was calculated as follows:

ECV=(administereddose−totalactivityeliminated)/plasmaconcentration

The result was given as mean value of each ECV_period_. In the case of urinary loss throughout the test, only first period(s) was/were considered in the calculation.

BSA was calculated according to the Haycok formula as recommended in guidelines of European association of nuclear medicine [16,17]. Measured GFR was scaled to standard ECFV (12.9 L) [18] or to standard BSA (1.73 m^2^) according to the individual values of ECFV or BSA.

Participants underwent either a Roux-en-Y gastric bypass (RYGB) or a sleeve gastrectomy (SG). All participants gave their written informed consent and the study was approved by our local ethic committee (“Sud Méditerranée” Institutional Review Board, ClinicalTrials.gov number 2014-A01208-39). Considering that BSA and ECFV in obese individuals both decreased by around 15% after BS [19], we estimated that mGFR_ECFV_ would increase by more than 20% in our participants. Since we observed a maximum standard deviation of 6 mL/min/12.9 L with our measurement technique in participants with severe or morbid obesity, we calculated that ten participants would be enough to identify a 20% increase of mGFR_ECFV_ between before and one-year after BS with an alpha risk of 5% and a power of 90%. Continuous variables are presented as means ±SD and categorical variables as numbers. The results before and after BS were compared using a paired bilateral Student’s t-test (distributions tested by Shapiro-Wilk test) for continuous variables and an Exact Fisher test for categorical variables. Statistical analyses were performed using SAS Enterprise Guide 7.1 (Copyright (c) 2017 by SAS Institute Inc., Cary, NC, USA).

## Results

We screened 1774 participants between October 2015 and February 2018. Forty-one had CKD stage 3 and 26 did not accept to enter the study or did not meet the criteria for BS [14]. We enrolled 15 participants, but five were excluded because their mGFR_BSA_ was above the threshold of 60 mL/min/1.73 m^2^ (four participants) or because a severe acute kidney injury occurred after BS (one participant). Ten participants completed the study. The characteristics of participants at inclusion are summarized in Table 1. Surgical procedures were SG in six participants and RYGB in four. Comparisons of variables measured before and after BS are shown in Table 2.

**Table 1:** Characteristics of the participants at baseline (n = 10).

Sex (men/women)	**5/5**
Age (years)	**56.7 ± 7.4**
Weight (kg)	**121.9 ± 20.1**
Height (cm)	**167.4 ± 9.8**
BMI (kg/m^2^)	**43.3 ± 3.6**
Waist circumference (cm)	**130.3 ± 16.8**
eGFR (mL/min/1.73m^2^)	**48 ± 8**
Edema	**8/10**
Type 2 Diabetes	**6/10**
Hypertension	**8/10**
Sleep apnoea syndrome	**6/10**
Disabling osteoarticular disease	**1/10**
Non-alcoholic fatty liver disease	**2/10**

eGFR = estimated glomerular filtration rate; BMI = body mass index.

**Table 2:** Changes of the mains parameters before and after bariatric surgery.

	Before BS	After BS
**Weight (kg)**	**122 ± 23**	**93 ± 20** **
**BMI (kg/m** ^**2**^ **)**	**43.3 ± 3.8**	**32.7 ± 4.3** **
**BSA (m** ^**2**^ **)**	**2.43 ± 0.28**	**2.10 ± 0.27** **
**24 h urinary creatinine (mmol/24h)**	**13.3 ± 3.8**	**10.8 ± 3.6**
**UACR (mg/g)**	**235 ± 437**	**177 ± 346**
**Plasma leptin (ng/mL)**	**74.8 ± 51.8**	**31.8 ± 39.3** *
**ECFV (L)**	**23.2 ± 6.2**	**17.9 ± 4.3** *
**Antihypertensive drugs/participant**	**1.5 ± 1.0**	**0.3 ± 0.5** *
**Physical signs of edema**	**8**	**4**
**Participants with insulin**	**3**	**0**

* Significant difference p<0.005;

** Significant difference p<10^−5^.

UACR = urinary albumin over creatinine ratio; ECFV = extracellular fluid volume; mGFR = measured glomerular filtration rate; BSA = body surface area; BMI = body mass index.

One year after BS, the mean excess BMI loss was 59 ± 19%, the mean change in plasma leptin levels was—43 ± 25 ng/mL (p = 0.004) and the mean change in urinary creatinine levels was—2.5 ± 3.5 mmol/24h (p = 0.052). The UACR was not significantly changed after BS (235 ± 437 versus 177 ± 346 mg/g, p = 0.157). Following weight loss (122 ± 23 kg versus 93 ± 20 kg, p < 0.001), BSA decreased (2.45 ± 0.29 versus 2.11 ± 0.28 m^2^, p < 0.001) by 14% and ECV decreased (23.2 ± 6.2 versus 17.9 ± 4.3 L, p = 0.001) by 23%. 60% of the participants had no more physical signs of edema after BS and the number of drugs required to control blood pressure was lower (1.5±1.0 versus 0.3±0.5, p < 0.001).

**Fig 1:**
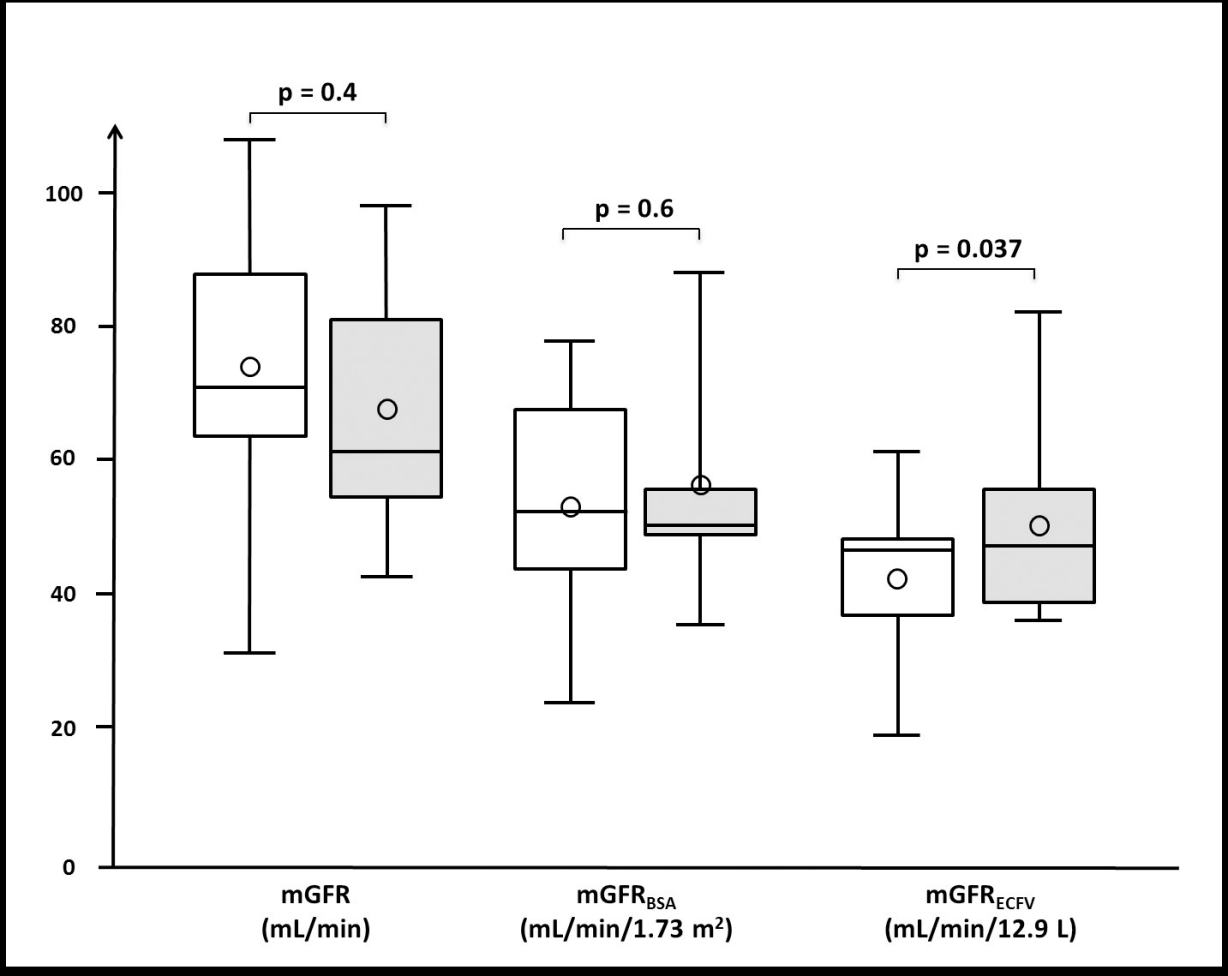
**Renal function before (white box) and after (grey box) bariatric surgery (BS).** The absolute measured glomerular filtration rate (mGFR, mL/min) and the mGFR scaled to standard body surface area (mGFR_BSA_, mL/min/1.73 m^2^) are not statistically different before and after BS. The mGFR scaled to standard extracellular fluid volume (mGFR_ECFV,_ mL/min/12.9 L) is significantly higher after BS. Box-plots stand for median and interquartile ranges (25–75). Circles stand for means, and extreme values are shown with lines. Statistics are obtained with paired Student’s t-tests.

**Fig 2:**
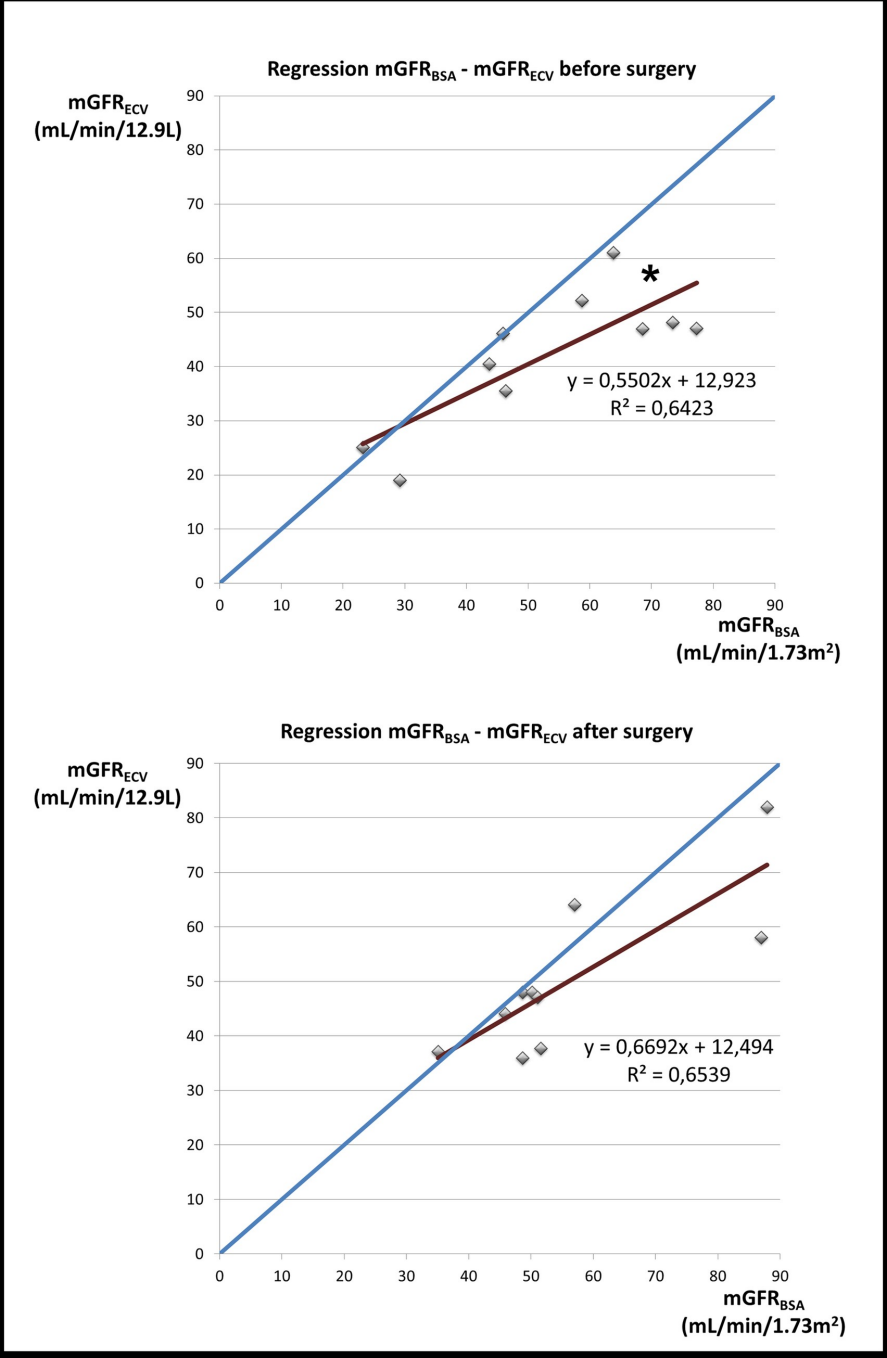
Change of relationship between mGFR_ECFV_ and mGFR_BSA_ after bariatric surgery. This figure shows the slopes of the relation between measured glomerular filtration rate (mGFR) scaled to standard extracellular fluid volume (mGFR_ECFV_) and mGFR scaled to standard body surface area (mGFR_BSA_). The slope is significantly different from the identity line before bariatric surgery (BS) (* p = 0.014). In contrast, the slope is no longer different from the identity line after BS (p = 0.09). Statistics are obtained with Student’s t-test.

BS neither significantly modified absolute mGFR (74 ± 23 versus 68 ±19 mL/min) nor mGFR_BSA_ (53 ± 18 versus 56 ± 17 mL/min/1.73 m^2^). In contrast, mGFR_ECFV_ increased significantly after BS (from 42 ± 13 to 50 ± 14 mL/min/12.9 L, p = 0.037) (Fig 1). Finally, the slope of the relationship between mGFR_ECFV_ and mGFR_BSA_ was different from the slope of the identity line before BS (p = 0.014); in contrast there was no longer a difference after BS (p = 0.09) (Fig 2).

## Discussion/Conclusion

This study shows that mGFR_ECFV_ increases one year after BS (shown in Fig 1). This is explained by the large decrease of ECFV and by the stability of the absolute mGFR in our participants, in line with the expected absence of renal functional reserve [7,8]. Indeed the reserve filtration capacity seems to be lost or attenuated when the kidney is already maximally stimulated, as this is the case in hyperfiltration due to morbid obesity. This lack of renal reserve and the absence significant changes in mGFR before and after bariatric surgery allow for easy comparison between BSA and ECFV as indexation variables.

From a physiological point of view, one of the aims of the kidneys is to get rid of waste products of metabolism from the ECFV whereas BSA is an arbitrary variable unrelated directly to kidney function. Based on this principle, this study confirms that ECFV better reflects the glomerular filtration demand than BSA. Indeed, BSA is not a substitute for ECFV in our participants with severe or morbid obesity because the relation between mGFR_ECFV_ and mGFR_BSA_ is not similar before and after BS in the same subjects (shown in Fig 2). Peters et al. already reached this conclusion for children [20] that ECFV is a physiologically valid variable to express GFR and is probably preferable to BSA. This finding in children can be applied to obese adults because of similar body shape. If human shape is considered to be cylindric, the shape of any individual can be expressed as the ratio of height to effective radius. From this equation, Bird et al. noticed that very small children with small BSA exhibit a similar height/effective radius as adults with a very large BSA [18]. Modifications of body shape following BS may be schematized by an increase of the ratio of height to effective radius, mimicking the evolution of body shape from childhood to adulthood. This analogy is noteworthy because only mGFR_ECFV_ properly describes the evolution of the renal function from childhood to adulthood whereas mGFR_BSA_ does not [18]. Finally, the use of BSA as an indexation variable for the renal function in obese individuals can be misleading [21] because BMI and BSA are no longer in a linear relationship over 35 kg/m^2^ [10].

Regarding body composition changes following BS, we observe that plasma leptin decreases significantly but not urinary creatinine excretion (shown in Table 2). This is in line with the more significant loss of fat mass than lean mass after BS. Actually, BS usually achieves a dramatic reduction of fat mass by 50% in comparison to a slight lean mass reduction of around 15% [22] and thereby restores the proportionate relationship between BMI and fat over lean mass ratio [23].

The elevation of mGFR_ECFV_ together with the reduction of the number of antihypertensive medications and the withdrawal of insulin following BS (shown in Table 2) might predict a possible nephroprotective effect of BS [24]. Proteinuria remains identical before and after BS, as already observed in a short-term study enrolling similar patients [8], disclosing unchanged intraglomerular pressure, or irreversible glomerular damage, or both (shown in Table 2). In contrast, microalbuminuria in participants with preserved renal function is usually reversible after BS, as shown in two meta-analyses [25,26]. This probably reflects the lower intraglomerular pressure achieved following BS because GFR and microalbuminuria decrease simultaneously [25,26]. Nevertheless, these individuals are not comparable with our participants with stage 3 CKD who are presenting with irreversible glomerular changes due to diverse causes. Therefore, the comparisons between mGFR or proteinuria in the short term seem to be inappropriate to assess a nephroprotective effect of BS in obese individuals with CKD. In an effort to have greater insight into renoprotection with BS over time, Chang et al. demonstrated an improvement in kidney outcomes after BS at five years [27]. Unfortunately, their long-term study used the estimated GFR based on serum creatinine instead of mGFR, which could have skewed analysis interpretation [6]. Finally, it would be relevant to take into account the duration of obesity prior to BS because obesity accelerates the decline of renal function in IgA nephropathy [28], after unilateral nephrectomy for cancer [29] or after renal transplantation [3]. All these questions raised by our pilot-study remain to be challenged by the measurement of the renal function trajectory over a longer period of time in randomized controlled trials in individuals with diverse duration of obesity [6].

The strengths of our study are its paired design and the use of mGFR which is not readily available in many hospitals. Limitations of this study include its small sample size, the measurement of lean body mass with 24 h urinary creatinine output and of fat body mass with the plasma levels of leptin. Actually, the gold standard methods to access body composition are dual-energy X-ray absorptiometry (DEXA) or air-displacement plethysmography (Bod Pod) [30]. However, the 24 h urinary creatinine output matches lean body mass even in patients with obesity [31], and leptin reflects fat mass [32].

In conclusion, we provide evidence that indexing mGFR by BSA or by ECFV does not lead to the same conclusions regarding the evolution of the renal function following BS. The use of mGFR scaled to ECFV seems physiologically more relevant and could be preferred to assess the nephroprotective effect of BS in ongoing or future clinical trials.

## Supporting information

S1 Table Data basis including all variables collected. (PDF)
