# Supplementary material for: Extracellular fluid volume: A suitable indexation variable to assess impact of bariatric surgery on glomerular filtration rate in patients with chronic kidney disease
Source: PLoS One. 2021 Aug 16;16(8):e0256234. doi: 10.1371/journal.pone.0256234 (PMC8366966; doi:10.1371/journal.pone.0256234)
Supplement: S1 Table — (PDF) [file pone.0256234.s001.pdf]

S1 Table. Data basis including all variables collected.

BEFORE SURGERY															
Patient N°	Weight *(kg)*	BMI *(kg/m^2)*	mGFR *(mL/min)*	ECFV *(L)*	$mGFR_{ECFV}$ *(ml/min/12.9L)*	BSA *(m^2)*	$mGFR_{BSA}$ *($mL/min/1.73m^2$)*	eGFR MDRD *($mL/min/1.73m^2$)*	Plasma leptin *(ng/mL)*	24h urinary creatinine *(mmol/24h)*	UACR *(mg/g)*	Number of antihypertensive drugs	Insulin treatment *(1 = yes, 0 = no)*	Physical signs of edema *(1 = yes, 0 = no)*	Surgery method
1	112.7	42.9	31	16.0	25	2.31	23	34	176.0	8.0	257	2	0	1	SG
2	139.5	44.5	68	21.7	40	2.69	44	50	38.0	19.7	168	3	1	1	SG
3	179	51.2	53	36.1	19	3.14	29	37	86.0	15.2	16	2	1	1	SG
5	112.5	41.3	86	18.2	61	2.33	64	60	93.0	16.1	6	0	0	1	RYGB
7	116.9	45.7	63	22.9	35	2.35	46	49	-	9.9	222	1	1	1	RYGB
8	122.3	43.3	65	18.2	46	2.45	46	42	54.0	16.8	206	2	0	0	SG
11	105	40.5	88	24.2	47	2.22	69	50	60.0	10.1	5	1	0	1	RYGB
12	124.6	40.7	107	28.7	48	2.52	73	57	13.4	12.6	25	2	0	0	SG
13	105.1	37.2	101	27.4	47	2.26	77	60	16.3	15.2	1444	2	0	1	RYGB
14	105.5	45.7	74	18.3	52	2.18	59	59	-	9.6	5	0	0	1	SG

AFTER SURGERY															
Patient N°	Weight *(kg)*	BMI *(kg/m^2)*	mGFR *(mL/min)*	ECFV *(L)*	$mGFR_{ECFV}$ *(ml/min/12.9L)*	BSA *(m^2)*	$mGFR_{BSA}$ *($mL/min/1.73m^2$)*	eGFR MDRD *($mL/min/1.73m^2$)*	Plasma leptin *(ng/mL)*	24h urinary creatinine *(mmol/24h)*	UACR *(mg/g)*	Number of antihypertensive drugs	Insulin treatment *(1 = yes, 0 = no)*	Physical signs of edema *(1 = yes, 0 = no)*	%EBMIL
1	90.9	34.2	42	14.6	37	2.07	35	28	118.0	10.7	330	1	0	0	48.6
2	100.5	32.1	75	15.1	64	2.25	57	47	10.5	14.7	50	1	0	1	63.6
3	140.2	40.1	82	28.1	38	2.75	52	48	19.2	8.9	55	0	0	1	42.4
5	73.6	27.0	94	14.8	82	1.85	88	75	11.9	10.1	2	0	0	0	87.7
7	79	29.7	54	19.4	36	1.92	49	57	7.4	9.8	45	0	0	1	77.3
8	110	39.4	65	17.7	48	2.31	49	59	26.8	18.7	113	1	0	0	21.3
11	86	33.2	58	15.6	48	2.00	50	52	33.1	5.6	22	0	0	0	47.1
12	90	29.4	56	16.4	44	2.11	46	61	-	11.4	23	0	0	0	72.0
13	81.5	29.2	98	22.0	58	1.97	87	78	3.2	9.1	1122	0	0	0	65.6
14	76	32.9	54	14.8	47	1.83	51	59	28.3	9.5	5	0	0	1	61.8

BMI = body mass index; mGFR = measured glomerular filtration rate; ECFV = extracellular fluid volume; $mGFR_{ECFV}$ = mGFR scaled to standard extracellular fluid volume; BSA = body surface area; $mGFR_{BSA}$ = mGFR scaled to standard body surface area; eGFR MDRD= estimated glomerular filtration rate with MDRD formula (Modification of Diet in Renal Diseases); UACR = urinary albumin over creatinine ratio; %EBMIL = percent of excess BMI loss; SG = sleeve gastrectomy; RYGB = Roux-en-Y gastric bypass.
